# A Technique of Predicting Radiographic Joint Line and Posterior Femoral Condylar Offset of the Knee

**DOI:** 10.1155/2014/121069

**Published:** 2014-02-11

**Authors:** Nicholas D. Clement, David F. Hamilton, Richard Burnett

**Affiliations:** Department of Orthopaedics and Trauma, The Royal Infirmary of Edinburgh, Little France, Edinburgh EH16 4SA, UK

## Abstract

*Purpose*. To describe a reliable method of predicting native joint line and posterior condylar offset (PCO) using true lateral digital radiographs of the distal femur. *Methods*. PCO was measured relative to a line drawn parallel to the posterior cortex of the distal femur and the joint line was measured from the posterior condylar flare to the articular surface. A ratio was then calculated for these measurements relative to the width of the femur at the level of the flare. Two independent observers measured PCO and joint line ratio for 105 radiographs of the different knees and one repeated these measurements after one week. *Results*. There was a significant correlation between the width of the femoral diaphysis at the level of the posterior condylar flare with joint line (*P* = 0.008) and PCO (*P* = 0.003). Joint line and PCO could be predicted within 4 mm and 2 mm, respectively, using the identified ratio between the width of the femoral diaphysis at the level of the posterior condylar flare with measured joint line and PCO. The inter- (*P* < 0.001) and intra- (*P* < 0.001) observer reliability for these ratios were high. *Conclusion*. These ratios could be used to predict the native joint line and PCO.

## 1. Introduction

The rate of total knee replacement (TKR) has increased rapidly during the last decade, and approximately 64,000 are performed each year in the UK [1]. The frequency of revision surgery has also increased, but at a greater rate, with more than double the number being performed now compared to the beginning of the decade [2]. This revision burden will likely continue to increase in the future due to the accelerating rate of primary TKR. It is acknowledged that the outcome of revision TKR is inferior to primary TKR [3].

Joint line position after primary TKR has been shown to correlate with functional outcome [4]. Failure to restore the joint line in revision TKR has also been demonstrated to result in a diminished functional outcome [5]. This may be related to increased patellofemoral joint contact forces, which increase with elevation of the joint line [6]. Due to distal femoral bone loss, elevation of the joint line in revision TKR may occur if distal femoral augments are not used [5, 7, 8].

Restoration of posterior femoral condylar offset (PCO) is an important aspect of TKR, providing flexion stability and range of movement [9–11]. However, restoration of PCO during revision TKR is difficult due to posterior femoral condylar bone loss and can result in undersizing of the femoral component [12]. Hence, to balance the knee in both flexion and extension, a thicker polyethylene insert will be needed, which will result in elevation of the joint line [12]. Whether loss of PCO affects the outcome of revision TKR remains unknown.

Recently, Johal et al. [13] described a method of measuring the PCO radiographically, describing a ratio of the PCO divided by the femoral diaphysis measured on a lateral radiograph of the knee. However, we are unaware of a radiographic method or tool that enables prediction of native joint line. If these two important measurements could be predicted, they would serve as a reference point to which the restored joint line and PCO could be compared after both complex primary and revision TKR, where measurement pre-operatively may not be accurate. The aim of this study was to assess the inter- and intraobserver reliability of a method to predict native joint line and PCO using digital radiographs.

## 2. Patients and Methods

During a one-week period at the study centre, 146 radiographs of the knee were performed. These radiographs were stored upon the local digital picture archiving and communication system. Twenty-eight were of a TKR and 13 radiographs did not include a true lateral radiograph of the distal femur. The remaining 105 radiographs of the native knees that included a true lateral (overlap of the posterior femoral condyles) and centred on the knee were defined as the study cohort. This cohort included 60 (57%) female and 45 (43%) male patients with a mean age of 58 years (range 25 to 86 years). The indications for the radiographs were multiple, younger patients generally had imaging to exclude a fracture after trauma, and older patients had imaging to assess degenerative change within the knee joint.

**Figure 1:**
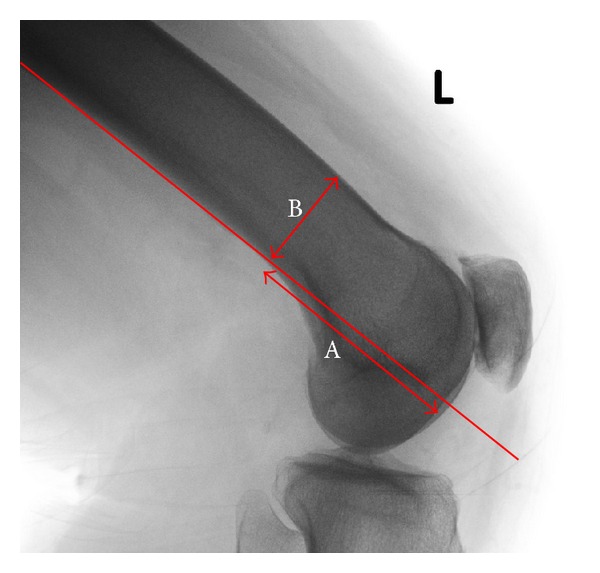
Measurement of joint line on the lateral radiograph to the distal femur. Joint line was measured (A) from the point at the tangent of the posterior cortex of the femur that crossed the flare of posterior condyles to the distal femur along that line. The joint line ratio was calculated by dividing the width of the femoral diaphysis at the level of the condylar flare (B) by the joint line (A/B).

**Figure 2:**
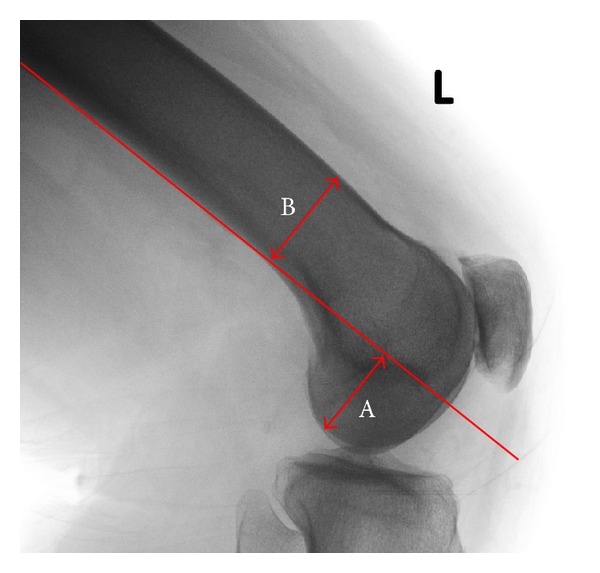
Measurement of PCO on the lateral radiograph to the distal femur. PCO was measured (A) relative to the tangent of the posterior cortex of the femur. The PCO ratio was calculated by dividing the width of the femoral diaphysis at the level of the condylar flare (B) by the PCO (A/B).

A modification of Bellemans et al. [9] technique for measuring PCO was used to measure both joint line and PCO as a ratio relative to the diameter of the femoral diaphysis. All measurements were made using the lateral radiograph of the distal femur. Joint line was measured from the point at which the “flare” of the posterior femoral condyles crosses a tangent to the posterior cortex of the femoral diaphysis to the distal femur along that line (Figure 1). PCO was measured as described by Bellemans et al. [9] (Figure 2). A ratio was then calculated for joint line and PC, to adjust for radiographic magnification, relative to width of the femoral diaphysis at the point at which the flare of the posterior femoral condyles crosses the tangent to the posterior cortex of the femoral diaphysis (Figures 1 and 2).

All measurements were made using the graphics tools available on Kodak picture archiving and communication system on a liquid crystal display. A custom work list was created in which the patient's details were removed. Two independent observers measured joint line, PCO, and width of the femoral diaphysis after training and demonstrations. These data were used to assess interobserver reliability. One observer repeated the measurements one week later of the same radiographs in a random order. These data were used to assess intraobserver reliability.

Statistical analysis was performed using Statistical Package for Social Sciences version 17.0 (SPSS Inc., Chicago, IL, USA). Pearson's correlation was used to assess the relationship between joint line and PCO with the width of the femoral diaphysis. An unpaired Student's *t*-test was used to compare radiographic measurement and ratios between genders. A single measure intraclass correlation coefficient was used for the quantification of inter- and intraobserver reliability. This correlation is calculated from the estimated variance of the components measured, analysing not only the correlation between the observers but also their agreement. Values greater than 0.75 indicate satisfactory reliability [14].

## 3. Results

**Figure 3:**
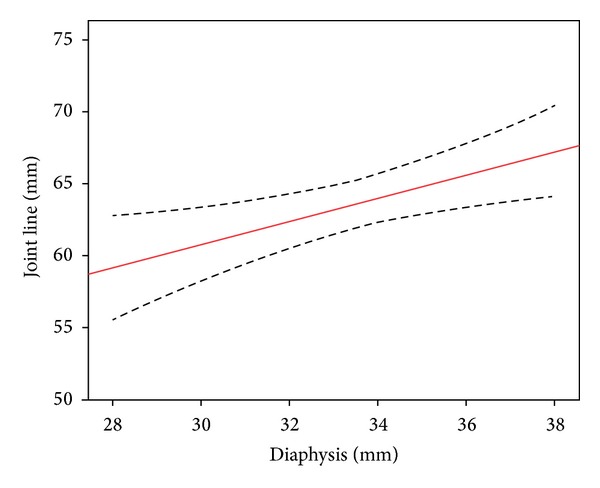
Correlation between joint line and the width of the femoral diaphysis (dashed lines represent 95% confidence limits).

**Figure 4:**
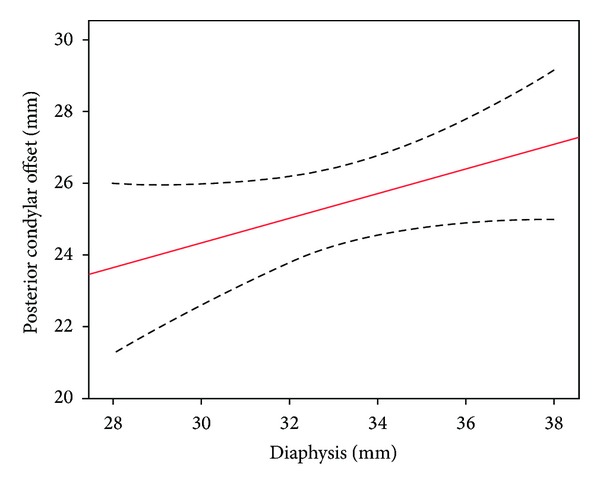
Correlation between PCO and the width of the femoral diaphysis (dashed lines represent 95% confidence limits).

Radiographic measurements were normally distributed (Table 1). The width of the femoral diaphysis correlated with joint line (*r* = 0.47, *P* = 0.008) and PCO (*r* = 0.4, *P* = 0.003) (Figures 3 and 4). Joint line ratio was 1.90 (95% confidence interval (CI) 1.85 to 1.96) and the ratio for PCO was 0.76 (95% CI 0.72 to 0.79). Using these ratios, it would be possible to predict the native joint line and PCO within 3.7 mm and 2.2 mm 95% of the time, respectively, assuming a mean of 33.4 mm for the width of the femoral diaphysis.

There was a significant difference in the measured joint line and width of the femoral diaphysis between genders, but this was not observed for PCO (Table 2). However, there was no significant difference in the ratios for joint line or PCO; hence it would seem that this adjusts for gender differences (Table 2).

The intraobserver reliability was high, with significant correlations being demonstrated for the radiographic measurements (Table 3). There was a greater correlation of the PCO ratio compared to joint line according to the intraobserver reliability. The interobserver reliability was also high, with significant correlations being observed for the radiographic measurements; however, the 95% CI was wider than that observed for intraobserver reliability (Table 3). Interestingly, after adjusting for radiographic magnification, there was a greater reliability between observers for the joint line and PCO ratios, with narrow 95% CI.

## 4. Discussion

This study has demonstrated a reliable method of predicting joint line and PCO using true lateral digital radiographs of the distal femur. There was a significant correlation between the width of the femoral diaphysis at the level of the posterior condylar flare with joint line and PCO. Joint line and PCO could be predicted within 4 mm and 2 mm, respectively, using the identified ratio between the width of the femoral diaphysis at the level of the posterior condylar flare with measured joint line and PCO. The inter- and intraobserver reliability were high, particularly between the ratios for the joint line and PCO. These ratios could be used to predict the native joint line and PCO to which postoperative radiographic assessment of complex primary and revision TKR could be compared to assess for the restoration native joint line and PCO.

A limitation of our study is the size of the chosen cohort with only 105 patients in total being radiographically assessed. In addition, there were various indications for the analysed radiographs with a broad age range. Despite the limited numbers and variable ages, the ratio of joint line and PCO relative to the width of the femoral diaphysis remained consistent, with good intra- and interobserver variability. This ratio may be dependent upon factors such as ethnicity, as the majority of the study population were composed of white UK nationals and further studies would be needed to conform the ratios across all ethnicities. This variation according to ethnicity was demonstrated in a recent study by Wang et al. [15], illustrating that the PCO in the Chinese population seems to be greater than that observed in a Western population. The ratio may also be different for children, but obviously this would not be necessary in revision arthroplasty.

Our results regarding the PCO ratio are supported by the findings of Johal et al. [13], who demonstrated similar measures for the PCO and the femoral diaphysis. They calculated their ratio differently from that described in the current study, but their PCO ratio was found to be 0.44 and when calculated using their methodology, our overall ratio is 0.43, demonstrating the reliability of the measurements in a Western population. However, in the recent study by Wang et al. [15] a significantly greater PCO ratio was demonstrated in the Chinese population when compared to a Western population of 0.47 in females and 0.46 in males. This supports the validity of the ratio but does illustrate that it varies according to the population assessed.

In contrast to Johal et al. [13], the current study used digital picture archiving to measure our radiographs, which are becoming more prevalent within medical healthcare [16]. Digital radiographs have several advantages over standard imaging, including a reduction in radiation exposure, fewer instances of over- and underexposure, and reduced rates of unsatisfactory films [17]. There is however an inherent error when making absolute measurements on digital radiographs due to differences in the magnification between radiographs performed at differing times and between patients. The ratio we therefore propose offers a simple index to measure joint line and PCO which is independent of radiographic magnification.

Restoration of the native joint line during revision of total knee surgery or complex primary TKR is difficult. Failure to restore this joint line results in a poor functional outcome for the patient. Porteous et al. [5] demonstrated a diminished functional outcome in patients undergoing revision TKR with elevation of the joint line more than 5 mm. Partington et al. [7] also showed that with elevation of the joint line after revision TKR resulted in a poorer functional outcome, but they found a greater tolerance of up to 8 mm. This variation may be due to the error associated with the radiographic measures of joint line, which may relate to magnification error. In addition, multiple radiographic methods of measuring joint line have been described, which may also be attributed to the error in the absolute measurement of the joint line [4, 5, 7, 18, 19]. Our described method of prediction of the native joint line radiographically offers an accurate technique, within 4 mm, to which postrevision TKR radiographs could be compared and is independent of magnification. Furthermore, this method of predicting joint line could be used for the complex primary TKR, where the bony anatomy is grossly eroded and degenerate, offering an accurate assessment of where the restored joint line should be.

Restoration of PCO after TKR is essential to provide stability in flexion and optimise the range of motion [9]. Distal femoral bone loss in revision TKR may result in the surgeon undersizing the femoral component and using a thicker polyethylene to balance the knee [12]. In addition, the use of intramedullary stems to provide implant stability influences the anteroposterior position of the condylar portion of the femoral component [20]. The use of straight stems has been demonstrated to result in a diminished PCO relative to offset stems [21]. PCO is an independent predictor of range of movement after TKR, with increasing PCO having a direct correlation with increasing range of movement [9, 10]. Whether this is the case after revision TKR remains unknown. Malviya et al. [10] demonstrated that after primary TKR, joint line was not a predictor of range of movement, but they found PCO to have a greater and more significant correlation. This raises the following question; is the joint line elevation of 5 mm or 8 mm defined by Porteous et al. [5] and Partington et al. [7], respectively, associated with a poor functional outcome or is it due to the associated diminished PCO? Using our ratio to predict the native PCO, it would enable a comparative measure with 2 mm to be made after revision TKR, which is also independent of radiographic magnification.

## 5. Conclusion

Using ratios relative to the fixed measure of the femoral diaphysis enables a comparative measure of the restored joint line and PCO to be made. This would facilitate preoperative planning, enabling the surgeon to predict if the joint line needs to be moved distally and also predict the size of the femoral component. Using the measure as a comparative guide, future research may reveal that joint line is not as important as restoration of PCO in an effort to improve the outcome of revision TKR.

## Figures and Tables

**Table 1:** The mean, standard deviation (SD), and range for all radiographic measurements.

Radiographic measure	Mean (mm)	SD	Range
Joint line	63.5	5.0	53 to 71
PCO	25.5	3.0	21 to 32
Femoral diaphysis	33.4	2.9	28 to 38

**Table 2:** A comparison of radiographic measurements and ratios for male and female genders.

	Male (*n* = 45)	Female (*n* = 60)	Difference	95% CI	*P* value*
Joint line (mm)	65.5 (4.5)	61.9 (4.8)	3.6	0.1 to 7.1	0.045
PCO (mm)	26.5 (3.1)	24.7 (2.8)	1.8	−0.5 to 4.0	0.12
Femoral diaphysis (mm)	35.5 (2.0)	31.8 (2.5)	3.6	1.9 to 5.4	<0.0001
Joint line ratio	1.85	1.95	0.1	−0.06 to 0.18	0.1
PCO ratio	0.75	0.78	0.03	−0.01 to 0.06	0.32

*Unpaired *t*-test.

**Table 3:** Intraclass correlation coefficient for intra- and interobserver reliability for the different radiographic measurements and ratios.

Measure	Intraobserver reliability			Interobserver reliability		
	*r*	95% CI	*P* value	*r*	95% CI	*P* value
Joint line	0.95	0.87 to 0.98	<0.001	0.83	0.09 to 0.95	<0.001
PCO	0.94	0.74 to 0.98	<0.001	0.84	0.1 to 0.95	<0.001
Femoral diaphysis	0.89	0.79 to 0.95	<0.001	0.81	0.02 to 0.95	<0.001
Joint line ratio	0.84	0.68 to 0.92	<0.001	0.93	0.85 to 0.96	<0.001
PCO ratio	0.90	0.63 to 0.96	<0.001	0.93	0.86 to 0.97	<0.001
